# The Role of the Transcriptional Regulation of Stromal Cells in Chronic Inflammation

**DOI:** 10.3390/biom5042723

**Published:** 2015-10-16

**Authors:** Alvaro Valin, José L. Pablos

**Affiliations:** Servicio de Reumatología, Instituto de Investigación Hospital 12 de Octubre, Avenida Andalucía S/N, Madrid 28041, Spain; E-Mail: jlpablos@h12o.es

**Keywords:** inflammation, stroma, fibroblast, transcription, cancer, arthritis, NF-κB, STAT, HIF-1α, AP-1

## Abstract

Chronic inflammation is a common process connecting pathologies that vary in their etiology and pathogenesis such as cancer, autoimmune diseases, and infections. The response of the immune system to tissue damage involves a carefully choreographed series of cellular interactions between immune and non-immune cells. In recent years, it has become clear that stromal resident cells have an essential role perpetuating the inflammatory environment and dictating in many cases the outcome of inflammatory based pathologies. Signal transduction pathways remain the main focus of study to understand how stimuli contribute to perpetuating the inflammatory response, mainly due to their potential role as therapeutic targets. However, molecular events orchestrated in the nucleus by transcription factors add additional levels of complexity and may be equally important for understanding the phenotypic differences of activated stromal components during the chronic inflammatory process. In this review, we focus on the contribution of transcription factors to the selective regulation of inducible proinflammatory genes, with special attention given to the regulation of the stromal fibroblastic cell function and response.

## 1. Introduction

Chronic inflammatory reactions are characterized by two main features: persistence and predilection for certain sites. An inflammatory process reflects the host’s principal immune response designed to eliminate exogenous, or abnormal endogenous compounds produced during tissue injury. In general, the innate immune response is initiated within minutes, and can be supported by the adaptive immune response. Both systems are able to resolve the inflammation within several days. The response promotes the optimal restoration of tissue structure and function, but must also rapidly fall under control in order to prevent over reaction that could result in irreversible damage [1]. Failure to clear the endanger elements or inefficient termination of the response could result in chronic inflammation, occasionally leading to increased morbidity due to the induction of immunosuppression [1,2].

The response of the immune system to tissue damage requires the functional interaction between immune and stromal resident cells. The stroma is composed of a complex and loosely organized network of multiple cell types embedded in an extracellular matrix that provides structural support and participates in the control of cellular signaling. The stroma is mainly composed of the following major cell types: fibroblasts, pericytes, smooth-muscle and endothelial cells, pre-adipocytes, and mesenchymal stem cells. Tissue resident cells such as fibroblasts help define the microanatomy and architecture of organs and provide the appropriate microenvironment in which specialized functions can occur, but also play an active role in governing the persistence of inflammatory diseases. Aberrant temporal and spatial expression of adhesion molecules, chemokines, cytokines and their receptors, partly mediated by components of the stroma, has been shown to lead to persistent leukocyte retention and survival in these inappropriately stable stromal cell microenvironments [3].

## 2. Stromal Fibroblast as Modulators of Chronic Inflammation

Stromal fibroblasts dictate in many cases the outcomes of inflammatory based pathologies [4]. Fibroblasts control and support normal tissue homeostasis participating in multiple biological processes such as deposition of the extracellular matrix (ECM), regulation of epithelial differentiation, wound healing and senescence. Chronically inflamed tissue damage promotes the expression of a proinflammatory signature in stromal fibroblasts, leading to a more rapid proliferation rate, enhanced collagen production, secretion of growth factors and other ECM modulators, as well as the activation of unique expression programs [5,6,7]. These features are partially maintained in culture, implying stable alterations in these cells. Permanent changes in expression patterns have been shown in synovial fibroblasts from patients with rheumatoid arthritis (RA) compared with fibroblasts from non-inflamed joints [3], as well as in stromal fibroblasts and stromal tissue associated with cancer [8,9].

**Figure 1:**
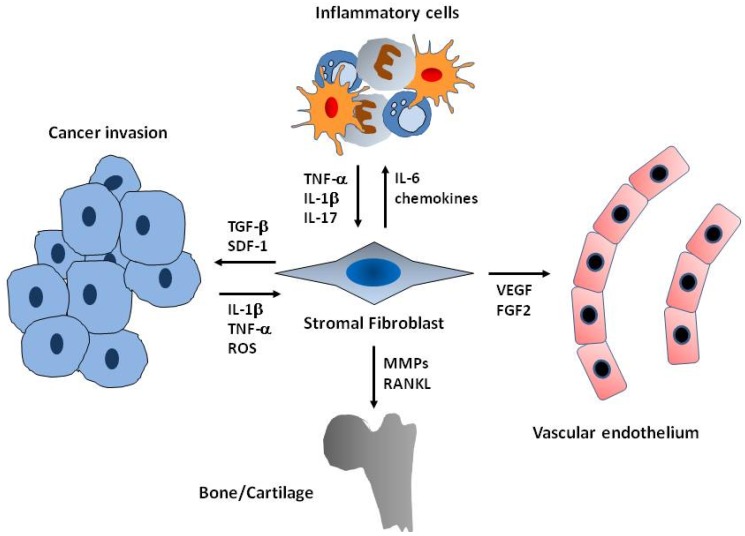
Functions of activated fibroblasts in the inflammatory stroma. Fibroblasts communicate with cancer cells, endothelial cells, and/or inflammatory cells through the secretion of cytokine, growth factors and chemokines. Both inflammatory and cancer cells activate resident fibroblast through the induction of cytokines and mediators such as IL-1β, TNF-α or ROS. In return, activated fibroblasts express additional cytokines and chemokines that recruit immune cells, perpetuating the inflammatory microenvironment. Fibroblasts interact with the microvasculature by secreting matrix metalloproteinases (MMPs) and pro-angiogenic factors such as VEGF or FGF-2. In addition, MMPs, RANKL and other matrix degrading factors expressed by fibroblasts enhance bone and cartilage destruction. Finally, stromal fibroblasts also provide potential oncogenic signals such as transforming growth factor-β (TGFβ) and SDF-1 (CXCL12) stimulating cancer-cell proliferation and invasion.

Investigations have shown a link between stromal fibroblasts and different pathology-related inflammatory processes in which common patterns of action emerge (Figure 1). First, fibroblasts-activating factors such as IL-1β and TNF-α are secreted by immune and/or tumor cells in the damaged tissue environment [10,11,12,13]. Following this activation, stromal cells initiate a proinflammatory response that includes the expression of interleukin-6 (IL-6) and IL-8 among others [12,13]. These secreted factors may modulate the pathological outcome in a direct manner, such as, for instance, increasing the proliferation rate of tumor cells, or sustaining the inflammatory environment by recruiting additional components of the immune system [10,11].

Rheumatoid arthritis synovial fibroblasts (RASFs) provide a clear example of how stromal fibroblasts contribute to the persistence of inflammation. RASF cells display an imprinted phenotype that is stable under *in vitro* culture conditions, reproducing functionally important effects such as cartilage invasion, as shown in severe combined immunodeficient (SCID) mouse models [14].

RASF-mediated erosion of cartilage and bone determines disease outcome for the majority of rheumatoid arthritis patients [15]. Furthermore, through secretion of cytokines and chemokines, synovial fibroblasts play a role in the persistence of inflammation in the synovium mediating the recruitment and retention of effector cells of the immune system [15,16]. Proinflammatory factors produced by immune cells and RASFs, such as IL-6, play a central role in the RA pathogenesis [17], actively contributing to inflammation, angiogenesis and matrix degradation [18,19].

Chronic inflammation enhanced by fibroblasts also strongly correlates with many types of human cancer. It has been shown that proinflammatory cancer-associated fibroblasts (CAFs) located within the tumor margins or infiltrated in the tumor mass express a proinflammatory gene signature in skin, breast, and pancreatic cancers among others [8,9,11]. CAFs have been shown to promote tumor growth by directly stimulating tumor cell proliferation and enhancing angiogenesis [20,21,22]. These secreted factors may affect tumor growth and metastasis in a direct manner or induce inflammation by recruiting components of the immune system [10,11]. Resident CAFs facilitate the transformation process [23] by secreting pro-tumorigenic factors as CXCL12 (SDF1) and TGF-β, expressing matrix metalloproteinases (MMPs) that alter the extracellular matrix composition and secreting proinflammatory cytokines such as IL-6 and IL-8 [12,13].

Many of the events displayed by pro-inflammatory fibroblasts are orchestrated at the nuclear level by a limited set of transcription factors that regulate the expression of specific gene programs. Under chronic inflammatory conditions, central signaling pathways including the transcription factors NF-κB, the STAT family of transcription factors, HIF-1α and AP-1 are activated [24,25]. These pathways have emerged as regulators of pro-inflammatory cytokines, angiogenesis, invasion, cell proliferation and survival, all involved in persistent inflammation.

## 3. Inflammation, Stroma, and the Sustained Inflammatory Environment

Cancer cells take advantage of the plastic nature of stromal and inflammatory cell populations, such as fibroblasts and macrophages, to generate a tumor enhancing microenvironment. A major tumor promoting mechanism is mediated through the production of cytokines by inflammatory and stromal cells that activate transcription factors in premalignant cells, particularly NF-κB and STAT3, but also AP-1, HIF-1α or Smads, giving rise to the expression of genes that stimulate cell proliferation and survival. NF-κB and STAT3 have been revealed as the two major transcription factors regulating the chronic inflammatory process in different pathologies. Both interact with each other at many different levels, amplifying their effect in feed forward loops that help to perpetuate the inflammatory environment. NF-κB and STAT3 are activated in the majority of inflammatory-based diseases and in cancer, where they are acting as non-classical oncogenes. However, their activation in pathological cells is rarely the result of direct mutations or mutational activation of upstream signaling components and instead depends on signals produced by neighboring immune and stromal cells. Both NF-κB and STAT3 mediated signals derived from tumor cells or infiltrating immune cells such as IL-1β, TNF-α, ROS or TLRs play a key role in the inflammatory activation of stromal fibroblasts associated to pathologies such as RA and cancer [10,11,12,13,26,27,28]. Pro-inflammatory fibroblasts have been shown to produce TNF-α, IL-1β, IL-6, cyclooxygenase-2 (COX-2), the polysaccharide hyaluronan, as well as inflammatory chemokines (e.g., IL-8, CCL5, CXCL1) [12,13,15], thus sustaining leukocyte recruitment into the inflamed tissue or supporting tumorigenesis and tumor-enhanced inflammation [10,11], activating genes that control cell survival, angiogenesis and invasiveness [24,28,29].

### 3.1. NF-κB Acts as a Master Regulator of Pro-Inflammatory Programs of Gene Expression

NF-κB has an important role in the activation of normal fibroblasts by immune and tumor cells [11]. Immune cells activate CAFs at the initial stages of tumorigenesis. Thus, for instance, during the early hyperplastic stage that leads to squamous cell carcinomas, the NF-κB dependent proinflammatory program in CAFs is induced by resident immune cells that have been stimulated by adaptive immune cells to express IL-1β [11,30]. In addition to IL-1β, a variety of stimuli could, in principle, give rise to the early premalignant expression of cytokines and chemokines in fibroblasts. These proinflammatory CAFs mediate innate immune cell recruitment and increase tumor angiogenesis, thereby enhancing tumor growth in an NF-κB-dependent manner.

Cancer cells can also activate fibroblasts. Co-culturing of pancreatic or lung cancer cells with stromal fibroblasts induced the expression of COX-2 and IL-8 [31,32], two factors associated to the stromal proinflammatory signature. Furthermore, in contrast to normal fibroblasts, that have a role in the maintenance of the epithelial homeostasis by suppressing their proliferation and oncogenic potential [21,33], following neoplastic transformation of epithelia, pro-inflammatory CAFs have been shown to promote tumor growth by inducing angiogenesis, recruiting bone marrow-derived endothelial progenitor cells and remodeling the ECM [20,22,34,35]. Similarly, primary skin fibroblasts from control mice incubated with conditioned medium of PDSC5 cells, an HPV16-derived skin carcinoma cell line, were promoted to induce the inflammatory signature [11]. Furthermore, purified CAFs from orthotopic tumors coinjected with normal skin fibroblasts expressed proinflammatory genes even though these fibroblasts were originally negative for the inflammatory gene signature [11].

NF-κB plays an essential role in the induction of the proinflammatory signature of CAF cells. The growth of tumors coinjected with shRNA IKKβ fibroblasts, where translocation of the NF-κB to the nucleus is inhibited, was significantly slower than in tumors coinjected with control fibroblasts. Furthermore, these tumors contained fewer infiltrating macrophages and were significantly less vascularized than controls. This NF-κB-induced proinflammatory gene signature is found in CAFs isolated from different tumors such as as skin, breast, and pancreatic cancers, suggesting a broader link between cancer and inflammation [11].

The presence of activated NF-κB transcription factors has also been demonstrated in cultured synovial fibroblasts (SF) [36], human arthritic joints [37,38], and the joints of animals with experimentally induced arthritis [39,40]. Immunohistochemistry has shown the presence of both NF-κB p50 and p65 subunits in the nuclei of cells lining the synovial membrane and macrophages [38,39]. Inhibition in synovial fibroblast of components of the NF-κB pathway profoundly inhibited the expression of proinflammatory factors as IL-6, IL-8 and VEGF [41], suggesting that NF-κB could be playing a major regulatory role in the production of inflammatory cytokine in pro-inflammatory fibroblasts.

The presence of activated NF-κB in SF can be the result of the continuous presence of inflammatory signaling and also their ineffective termination. Experiments with transgenic mice that express TNF-α suggest that SF are the major responders to TNF-α [42]. TNF-α stimulation of SF, in contrast to the effect in macrophages which are the main TNF-α producers and are located in close proximity to SF, resulted in a sustained inflammatory response characterized by prolonged expression of cytokines, chemokines, and matrix metalloproteinases (MMPs), associated with sustained NF-κB signaling and transcriptional activity and the ineffective induction of feedback mechanisms that contribute to the persistence of synovial inflammation [26].

**Figure 2:**
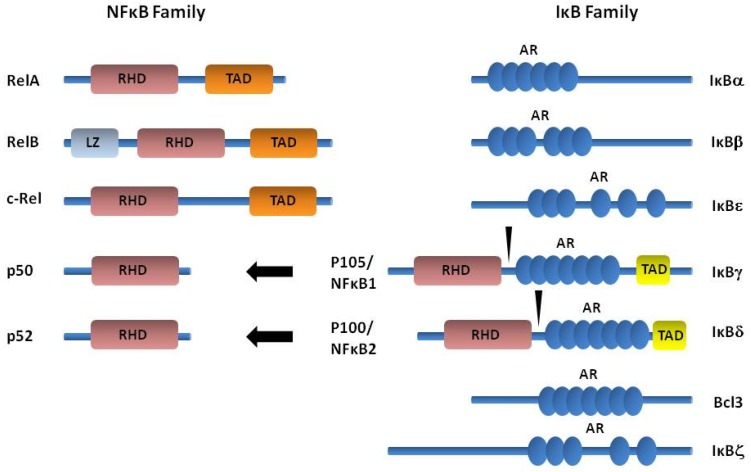
Members of the nuclear factor-κB (NF-κB) and IκB protein families. NF-κB proteins are defined by a *C*-terminal RHD, which can be activated by phosphorylation. p65, RelB and c-Rel contain TAD, which can activate NF-κB target genes. p100 and p105 lack TADs but contain AR domains and N-terminal DDs. RelB also contains a LZ. IκB proteins are characterized by the presence of ankyrin repeat domain (AR), shown as blue circles. NF-κB1/p105 and NF-κB2/p100 are precursor proteins, which give rise upon processing to p50 and p52. Their *C*-terminal region are ankyrin repeats analogous to those of the smaller IκBs. AR: ankyrin repeat; LZ: Leucine zipper domain; RHD: Rel homology domain; TAD: Transactivation domains.

The NF-κB family consists of five members: p105 (constitutively processed to p50), p100 (processed to p52 under tightly regulated conditions), p65 (also known as RelA), RelB and c-Rel [43] (Figure 2). The Rel homology region (RHR) that defines the NF-κB family supports sequence-specific DNA binding and the formation of stable homodimers and heterodimers. RelA, c-Rel, and RelB contain *C*-terminal activation domains, whereas p50 and p52 lack definable activation domains. In a broader scope, the studies described in NF-κB deficient mice have been important in establishing the molecular events that can occur in the NF-κB pathway, but also the potential role of stromal cells in inflammation. Mice with genetic deficiency of the genes encoding p50, p52, c-Rel and RelB develop normally. However, they have abnormal immune cell responses such as in B and T cell proliferation, antigen presentation, isotype switching, and cytokine production. Interestingly, mice with a targeted disruption of RelB developed a complex inflammatory phenotype and hematopoietic abnormalities [44,45] that may involve impaired regulatory T-cell function resulting from a combination of stromal and hemopoietic defects [46].

**Figure 3:**
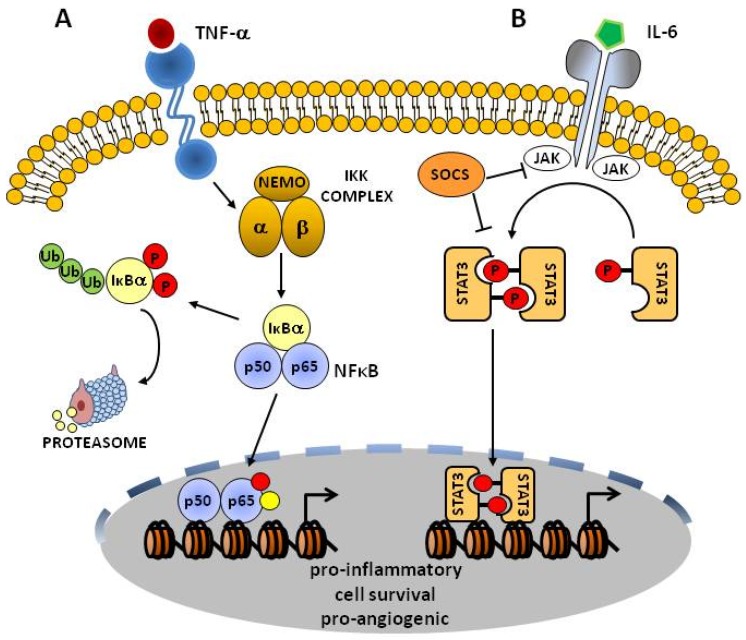
The NF-κB and STAT3 signaling pathway. (**A**) TNF-α is one of the most potent activators of NF-κB. TNF-α binds to the TNF-α receptor and activates the IKK kinase complex, which consists of three subunits: IKKα, β and Nemo (IKKγ). IKK phosphorylates (red circles) the inhibitory protein IκBα and targets this protein for Ub mediated degradation. The liberated NF-κB dimer translocates into the nucleus, undergoes additional post-translational modifications such as phosphorylation (red circle) and/or acetylation (yellow circle), and binds to κB elements in the promoters of target genes to regulate their expression; (**B**) Factors such as IL-6 signals through the JAK/STAT3 pathway. The IL6 receptor (gp130) activates intracellular JAK kinases, predominantly JAK2, which phosphorylate STAT3. Once phosphorylated, STAT3 molecules dimerize, translocate to the nucleus and bind to STAT response elements in the promoters of target genes. STAT3 signaling is tightly regulated by several inhibitory molecules, including suppressor of cytokine signaling (SOCS) proteins.

Most NF-κB proteins are retained in the cytoplasm of resting cells by ankyrin repeat-containing IκB proteins [43,47] (Figure 3A). Activation of the IκB kinase (IKK) complex phosphorylates IκBα and IκBβ and targets them for degradation by the ubiquitin/proteasome pathway, thus releasing NF-κB that translocate to the nucleus (Figure 3A). The IKK complex is regulated by multiple signals triggered by different cellular stimuli such as the bacterial endotoxin lipopolysaccharide (LPS) or cytokines such as TNF-α and IL-1, and thus playing an important role in the activation of NF-κB.

The molecular events leading to activation of NF-κB transcription factor in the RA synovium involve the three main players of the pathway, the IKK complex, IκBs and the NF-κB transcription factors itself. However, their contribution to the activation of NF-κB seen in RA may vary depending on the cell type and in response to different cellular stimuli. Dominant negative (dn) variants of these signaling components were expressed in cells relevant to RA, including primary synovial cell cultures (containing a mixture of cells) from patients undergoing knee replacement surgery, synovial fibroblasts derived from them, and primary M-CSF differentiated macrophages from normal human blood donors [41]. The IKK complex consists of at least three subunits: IKK1 (also known as IKKα), IKK2 (also known as IKKβ) and NF-κB essential modulator (NEMO, also known as IKKγ) (Figure 3A), and in these studies dnIKK1 was found not to influence spontaneous cytokine production from primary synovial cell cultures, whereas dnIκBα and dnIKK2 profoundly inhibited IL-6, IL-8 and VEGF production [41]. Dominant negative dnIKK2 was also found to inhibit cytokine production from both TNF-α and IL-1β stimulated macrophages and RASF fibroblasts, as well as IL-6 and IL-8 production in LPS stimulated RASF cells, supporting the idea of an important role for IKK2 in RA. In contrast to RASF fibroblasts, dnIKK2 did not affect TNF-α, IL-6 or IL-8 production upon LPS stimulation of human macrophages [41] although dnIκBα efficiently blocked their expression [41], suggesting that the NF-κB pathway is also activated in macrophages upon LPS stimulation. These studies support a differential contribution of the NF-κB signaling components and highlight the complexities of the role that the NF-κB pathway plays in RA.

### 3.2. The STAT3 Pathway

Similar to NF-κB, STAT proteins also regulate many aspects of growth, survival and differentiation in cells. The Janus kinase (JAK)-signal transducer and activator of transcription (STAT) pathway was originally discovered in the context of interferon-α (IFNα)-, IFNγ- and intereukin-6 (IL-6)-mediated downstream signaling [29] (Figure 3B). Of the seven members of the STAT protein family, STAT1, STAT3 and STAT5 have been demonstrated to be the most important for chronic inflammation and cancer progression [29,48]. Among them, an important feature of STAT3 is its crucial role in stromal cells, including immune cells, which are recruited to tumor microenvironments to promote cancer progression [29,49,50,51].

The inappropriate activation of STAT3 signaling pathways in tumor cells, alike NF-κB persistent activation, is not directly attributable to activating mutations in the genes encoding these transcription factors or the JAK/STAT pathway, although mutations in components of the signaling pathway such as gp130 and SOCS3, have been described in inflammatory liver tumours [52] and in lung cancer [53], respectively, resulting in STAT3 hyperactivation. However, the most common mechanism by which STAT3 transcriptional programs are induced is through an excess of activating cytokines provided in an autocrine or paracrine manner [54]. It was observed that many tumor cells, which display constitutive STAT3 activation *in vivo,* rapidly lose STAT3 phosphorylation once put into culture without neighboring immune or stromal cells [29]. Important cytokines and mediators involved in the induction and perpetuation of the inflammatory environment in cancer, such as IL-6, IL-1β, macrophage colony-stimulating factor (M-CSF) and cyclooxygenase 2 (Cox2), have the transcription factor STAT3 as a crucial regulator of their expression [29,55]. Although tumor cells are known to produce some of these mediators, they are mainly produced by the stromal inflammatory cells [29,49,50,51,56,57]. Importantly, the persistent activation of STAT3 intrinsic to tumor cells is transmitted to stromal inflammatory cells in the tumor microenvironment [58,59] through activation of cytokines, chemokines and growth factors, and associated receptors, which in turn activate STAT3 in stromal cells [29,60,61]. Therefore, STAT3 feed forward loops are established between tumor cells and non-transformed cells in the microenvironment, including immune cells [29].

Autocrine and paracrine feed forward loops formed by cytokine-STAT3 signaling are recurrent themes in many human cancers [29,57,62]. Thus for instance, STAT3 is a direct transcription factor for the sphingosine-1-phosphate receptor 1 (S1PR1) gene promoter, a G-protein-coupled receptors for sphingosine-1-phosphate (S1P), a biologically active metabolite of sphingolipid with critical roles in lymphocyte egress and chemotaxis, cell proliferation, survival, and tumor angiogenesis and metastasis [63]. In malignant cells and immune cells, but also in tumour stromal components such as endothelial cells, the expression of S1PR1 is upregulated [63,64,65]. STAT3-mediated S1PR1 upregulation, facilitated by sphingosine-1-phosphate (S1P) and IL-6, contributes in turn to sustain STAT3 activity in both tumor cells and in the tumor stromal cells, thereby promoting malignant progression.

Although a large number of growth factors and cytokines can stimulate STAT3 activity, which could have synergistic effects on prolonging STAT3 activation, many growth factors, cytokines and other factors that induce STAT3 activity in inflammation and cancer require the IL-6 signaling pathway [62]. IL-6 is a major inflammatory mediator and its uncontrolled production leads to chronic inflammation such as RA, inflammatory bowel disease, multiple sclerosis, and also many types of cancer. Interleukin-6, acting via STAT3 and STAT1, plays pivotal roles in governing leukocyte infiltration during acute inflammation [66,67,68] that may relate to the involvement of IL-6 in antimicrobial host defense and the inability of Il6−/− mice to effectively clear bacterial or viral infections [68,69,70]. However, inflammatory models of chronic disease and clinical observations identify IL-6 activity as detrimental in autoimmunity and cancer [67,69,70]. Thus, for instance, high levels of IL-6 and its soluble receptor IL-6R in synovial fluids of patients with RA and juvenile RA are associated with joint destruction and disease progression [71]. IL-6 deficiency resulted in complete protection against collagen-induced arthritis (CIA) in mice [72] and the anti-IL-6R monoclonal antibody Tocilizumab is an effective therapy for human RA [73].

Aberrant IL-6-Jak-STAT3 signaling in cancer cells has also emerged as an important mechanism for cancer initiation, development and progression [29,52,62,74,75]. Dysregulated production of IL-6 and aberrant IL-6 activation pathways have been reported in many human cancers and play important roles in various tumor behaviors such as proliferation, migration and adhesion [76]. Knockout gp130-757F/F mice, which carry a Y757F point mutation that disrupts the binding of the negative regulators SOCS3 and SHP2 to gp130, show hyperactivation of STAT3, resulting in chronic gastric inflammation and distal stomach tumors [77]. This IL-6-JAK-STAT signaling plays an important role in various tumorigenesis models, including breast, colon, lung, ovarian, prostate cancer, and multiple myeloma [62,78,79].

In addition to its direct importance to tumor cells, it has been demonstrated a major role of paracrine and autocrine IL-6/STAT3 signaling mediated by cells of the tumor microenvironment in facilitating tumor progression and inflammatory cell-mediated transformation [50,57,62,74,75]. Thus for instance, CAFs produced from liver metastases and normal liver fibroblasts are both able to induce IL-6 [80]. In addition to fibroblast, IL-6 secreted from other stromal cell types such as adipose cells, can promote migration and invasion of tumor cells such as breast cancer [81]. Adipose stromal cells (ASCs) significantly stimulate migration and invasion of ER-negative breast cancer cells *in vitro* and tumor invasion in a co-transplant xenograft mouse model. Depletion of IL-6 from the ASC conditioned medium abrogated the stimulatory effect of ASCs on the migration and invasion of breast tumor cells [81].

### 3.3. STAT3 and NF-κB Cooperate to Sustain Inflammation

STAT3 and NF-κB stimulate a highly overlapping repertoire of prosurvival, proliferative, and proangiogenic genes [24,29,74,82], and can cooperate at many levels. Thus, members of NF-κB like RelA can physiologically interact with STAT3 and their association can modify their transcriptional activity [83]. Stat3 interaction with RelA leads, for instance, to upregulation of the immunosuppressive IL-23/p19 gene [56]. NF-κB and STAT3 can cooperatively bind at a subset of gene promoters to synergistically induce their target genes expression [84]. In addition, many cytokines such as IL-6 expressed by NF-κB or STAT3 can feedback to induce in turn the activation of both transcription factors [62,78,85]. Through their functional interaction, NF-κB and STAT3 collaboratively promote tumor development via induction of pro-tumorigenic genes including genes in angiogenesis and hypoxia, chemokines and immunosuppressive cytokines [24,28].

Positive-feedback loop mediated by both NF-κB and STAT3 transcription factors may be important for the development of autoimmune diseases [86,87]. Thus, IL-6 together with IL-17A triggered a positive-feedback loop of IL-6 expression through the activation of NF-κB and STAT3 in fibroblasts. In F759 knock-in mice lines expressing mutated variants of the IL-6 signaling transducer gp130 [88], both IL-6- and gp130-mediated STAT3 activation are enhanced, developing a RA-like disease that depends on mature lymphocytes [89]. It was shown that the disease severity in the F759 mice is also accelerated in a manner dependent on IL-6 when the mice was crossed with human T cell leukemia virus 1 (HTLV-1) p40-Tax transgenic mice in which NF-κB signaling was enhanced [90]. These results suggest that IL-6 is one of critical factors for the rheumatic disease in F759 mice.

IL-6 is required for the development of Th17 cells, which are a subset of Th cells that express IL-17A [91,92,93] and cause chronic inflammation during autoimmune disease and transplant rejection [86,94,95]. IL-6 not only functions upstream of IL-17A but also acts as a critical downstream target of IL-17A. Inhibition of the IL-6 loop significantly suppresses the development of arthritis in F759 mice and in experimental autoimmune encephalomyelitis (EAE). IL-17A and IL-6 also synergistically induced the expression of various NF-κB target genes, including chemokines as CCL20, CXCL1, CXCL2, KC, MIP2, and IκB-z [86,87]. These investigations highlighted the central role of the enhanced signaling mediated through the IL-17A-triggered positive-feedback loop of IL-6 expression in fibroblasts in the development of arthritis in F759 mice. In this context, under pathological conditions in which Th17 cells trigger an autoimmune disease, the dysregulated enhancement mediated by the amplifier may be induced by unchecked activation of NF-κB and/or STAT3 in fibroblasts via a variety of environmental and/or genetic factors [86].

Other proinflammatory factors can potentially trigger NF-κB/STAT mediated loops similar to the IL-6/IL-17A feedback loop, contributing to sustaining the inflammatory network. Thus, *in vivo* evidence from DNase II-null, tumor necrosis factor (TNF-α)-transgenic, and TNF-α ARE mice suggests that systemically elevated levels of TNF-α induce chronic synovitis [42,96,97,98], mainly mediated through activation of stromal SF [42]. TNF-α can induce an unremitting inflammatory response in arthritic SF, which is characterized by sustained activation of the classic NF-κB pathway and continuous transcription of pathogenic mediators [26]. For genes that contain kappa-B (κB) motifs and STAT-binding sites, TNF-α-induced NF-κB and IFN-induced STAT work cooperatively by binding to promoters at the same time [99]. Prolonged TNF-α exposure open the chromatin structure, and enhanced the recruitment of NF-κB p65 and Pol II to the CXCL10/IP10 promoter. In parallel, an increase in intracellular STAT1 led to amplification of IFN-induced STAT1 activation. Chronic exposure of SF to TNF-α depletes histones and hyperacetylates the remaining histones, leading to loosening of chromatin at the locus of chromosome 4, where CXCL10, CXCL9, and CXCL11 genes are located in tandem, and enhancing the magnitude and extension of their expression upon subsequent IFN-induced STAT1 stimulation. Open chromatin in this specific location allows the unopposed recruitment of p65, STAT1 and Pol II, and provides the molecular basis for the gene-specific synergy [100].

### 3.4. Post-Translational Modifications Modulate Transcription Factors Activities

Post-translational modification of these transcription factors allows the precise regulation of expression in response to different signals. For instance, the interaction between phosphorylated and/or unphosphorylated forms of STAT3 and NF-κB have been reported previously by several groups. Unphosphorylated STAT3 (u-STAT3) forms a complex with the p65 subunit of phosphorylated NF-κB (p-NF-κB) on a κB sequence in the human IL-8 promoter, inducing gene expression in response to IL-1β [101]. U-STAT3 binds to both NF-κB p65 and p50 and a specific type of κB sequence motif supports both the binding of p65 homodimers and cooperativity with u-STAT3 [101]. In this regard, p-NF-κB synergistically cooperates with p-STAT3 and C/EBPβ to enhance transcription of the C reactive Protein (*CRP*) gene [102], and p-STAT3 and phosphorylated p65 form a complex following stimulation of cells with both IL-1 and IL-6, after which STAT3 interacts with non-consensus sequences at the 3' boundary of κB element of the serum amyloid A (*SAA*) promoter to enhance transcription [103].

The acute-phase reactant *SAA* is expressed in rheumatoid synovial tissues and induces the production of cytokines or chemokines in rheumatoid synoviocytes [104], supporting a pathogenic role of SAA in RA. The expression of IL-6 in response to activation of NF-κB by IL-1 initiates a positive feedback loop in which secreted IL-6 stimulates the tyrosine phosphorylation of STAT3, leading secondarily to an increase in u-STAT3, which then drives the expression of a subset of NF-κB-activated genes, including the chemokine CCL5/RANTES but also IL6, IL8, MET, and MRAS, that do not respond directly to p-STAT3 [84].

Many u-STAT3-responsive genes have κB elements that are activated by a transcription factor complex formed when u-STAT3 binds to unphosphorylated NF-κB (u-NF-κB), in competition with IκB. Thus, the κB element of the CCL5/RANTES promoter can function to give strong expression in two ways, directly in response to TNF-α or IL-1, or indirectly in response to IL-6 [84]. This dual regulation of CCL5/RANTES transcription may be important in regulating its physiological functions, with short-term expression in response to TNF-α or IL-1 controlled by p-NF-κB, and a more sustained expression in response to IL-6 regulated by u-STAT3:p-NF-κB [84]. This idea could also be relevant to explaining the sustained inflammatory response observed, for instance, in RASF cells, characterized by their prolonged expression of cytokines, chemokines, and matrix metalloproteinases (MMPs). Thus, in addition to other mechanisms described above, the sustained transcription observed in TNF-α-stimulated RASF cells of CCL5/RANTES, CXCL8/IL-8 or matrix metalloproteinases-1 (MMP-1) and MMP-3, could be the results of the differential response to phosphorylated NF-κB and STAT3 transcription factors.

## 4. Inflammation, Stroma, and the Innate Immune Response

The innate immune system is the major contributor to acute inflammation induced by microbial infection or tissue damage [105]. Activation of the innate immune system is characterized by the detection of pathogens via pattern-recognition receptors (PRRs) which trigger an inflammatory response. They do this by recognizing structures conserved among microbial species, which are called pathogen-associated molecular patterns (PAMPs), but also endogenous molecules from tissue damage called damage associated molecular patterns (DAMPs). Activation of the innate immune system by both types of stimuli play a role in inflammatory pathologies such as RA [106,107].

As a result of the inflammation in RA, endogenous ligands from tissue breakdown during joint destruction and present soon after matrix damage stimulate innate immune reactions in a positive feedback mechanism. The inflamed joint in RA is a source of many potential PRR ligands including HSP, fibrinogen, and hyaluronan. Four different classes of PRR families have been identified [108]. These families include transmembrane proteins such as the Toll-like receptors (TLRs) and C-type lectin receptors (CLRs), as well as cytoplasmic proteins such as the Retinoic acid-inducible gene (RIG)-I-like receptors (RLRs) and NOD-like receptors (NLRs). These PRRs act as extracellular and intracellular sensors of the innate immune system, responding to danger signals or pathogen components. Although innate immune cells including macrophages and dendritic cells (DCs) play important roles, stromal cells such as endothelial cells and fibroblasts also contribute to innate immunity. The sensing of PAMPs or DAMPs by PRRs in these cells generally upregulates the transcription of genes involved in inflammatory responses such as proinflammatory cytokines, type I interferons (IFNs), chemokines, antimicrobial proteins and proteins involved in the modulation of PRR signaling, although the expression patterns of the inducible genes differ among activated PRRs.

Current evidences suggest that fibroblast at the synovium act as effector cells of innate immunity. Bacterial products such as lipopolysaccharide (LPS) or peptidoglycan are known to activate FLSs by interacting with PRRs present on these cells [109,110], and ligands for PRRs such as the NOD-2 ligand MDP or bacterial peptidoglycans have been identified in the joints of patients with RA [111,112]. A number of TLRs and NLRs are detected in SFs, and their expression is increased in response to inflammatory stimuli (Table 1) [107,113]. Although SF expresses mRNA for TLRs 1-6, the predominant functional TLRs appear to be TLR2, 3 and 4 [107,114], detected in the synovium of patients with longstanding RA. Activation in RASF cells of TLRs by ligands such as those mentioned above and more likely by other yet unknown endogenous molecules may contribute to the perpetuation of inflammation and matrix destruction.

Signals from TLRs generally converge to activate the mitogen-activated protein kinases (MAPKs), the NF-κB pathway and interferon regulatory factor 3 (IRF3)/IRF7 pathways, which mediate the expression of inflammatory cytokines and type I interferon secretion, thus controlling the response to danger signals [108]. Stimulation of TLR2 signaling pathway in RASF leads to translocation of NF-κB, secretion of proinflammatory cytokines, matrix metalloproteases (MMPs) and expression of various chemokines [109,113,115,116]. Likewise, stimulation of TLR3 and TLR4 pathways by synthetic or endogenous ligands induces the production of interferon-β (IFN-β), IL-6, and the chemokines CXCL10 and CCL5 [117]. Cytokine and MMPs released by SF in culture can be rapidly upregulated by LPS and other TLR ligands [118] and the production of key mediators including RANKL appears to be dependent on both TLR2 and TLR4 signalling [119].

**Table 1:** PRRs in RASF cells.

Receptor	Ligands	Events	References
TLR2, TLR3, TLR4	Lipoproteins, glycolipids, dsRNA, LPS, poly I:C, heat shock proteins (e.g., fibrinogen, heparin sulphate, hyaluronic acid fragments)	Increase IL-6, MMP3 and MMP13 expression. Induction of IL-32β, γ, and δ mRNA. Enhanced osteoclastogenesis. mediated by increased expression of RANKL.	[107,119,120]
NOD1, NOD2	Products from gram-negative bacteria (e.g., diaminopimelic acids), muramyl dipeptide (MDP)	Stimulation of NOD1 upon induction of TLR3. NOD1 and TLR2 stimulates the expression of IL-6 synergistically. Production of IL-8, IL-6 and MMPs.	[121,122]
RIG-1, MDA5	dsRNA	Induction of CD55 expression and increased binding to CD97. MDA5 dose-dependent induction of cell death.	[114]

In addition to TLRs, SF expresses other PRRs, such as the RLRs members MDA5 and RIG-I [107,117,123,124,125]. Activation of TLR3 and RIG-I induces the expression of type I IFNs, cytokines, chemokines, and matrix metalloproteinases, mediated by activation of the transcription factors activator protein 1 (AP-1), NF-κB, and both IRF3 and IRF7, which are key factors in dsRNA sensor-mediated gene expression [108]. Activation of the dsRNA sensors TLR3, MDA5, and RIG-I robustly enhances the expression of CD55 in FLS. CD55 is known to strongly bind to CD97 expressed on most leukocytes, suggesting a possible local mechanism whereby an innate immune response could activate fibroblasts to promote leukocyte retention in the synovium [114].

Nod-like receptors NOD-1 and NOD-2 are also detected in RASF cells [121]. NOD-1 and NOD-2 are strongly expressed in RA synovium, leading to a rapid increase in the production of proinflammatory cytokines and MMPs, via MAPK and NF-κB signaling pathways [121,122]. NODs and TLRs can have synergistic effects [126,127]. NOD-1 expression is induced in RASFs upon stimulation of TLR3, potentially mediated by endogenous double-stranded RNA from necrotic cells [117]. In addition, NOD-1 synergize with the stimulatory effects of TLR2 and TLR4 in the production of IL-6 in RASF cells. Thus NLRs, either alone or through interactions with other inflammatory mediators, play important roles in the chronic and destructive joint inflammation in RA.

In addition to the role of the NF-κB pathway, IRF3 transcription factor seems to play a relevant role in the innate immune response of RASF cells. Transcription factors IRF3 and IRF7 bind to the IFN-stimulated response element (ISRE) and regulate transcription of IFN-stimulated genes that are expressed in rheumatoid joints, including IFN-β, RANTES, and IP-10 [128,129,130]. IFN-β protein is highly expressed in the synovium of patients with RA [128] and the gene expression profile in RA synovium displays characteristic features of the type I IFN signature [128,129,130]. The specific ligands or cytokines that activate the type I IFN response in RA synovium is not fully characterized although the activation of the TLR3 signaling pathway by virus infection, as well as endogenous ligands such as RNA released from necrotic cells in the synovial fluid could be participating [116,117,131]. The synthetic TLR3 ligand poly (I:C) and dsRNA associated with viral infections induces the IKK-related kinase IKKε, resulting in phosphorylation, nuclear translocation and dose-dependent promoter binding of IRF3, IRF7, NF-κB, and c-Jun/AP-1 in cultured RASF [132,133]. IRF3 phosphorylation is significantly increased in RA compared with osteoarthritis synovial tissue. In contrast to other poly (I:C) responsive cells as MEFs and bone marrow-derived cells where IRF7 is essential, IRF3 is the dominant transcription factor in primary human RA synoviocytes, whereas the contribution of IRF7 is relatively modest, suggesting a cell and/or ligand specificity in the type I IFN response.

In summary, PRRs’ activation by PAMPs and DAMPs regulate RASF activation and function. Although virus exposure or infection could have a role, endogenous ligands such as necrotic debris known to be present in the rheumatoid joint may contribute to synovial inflammation.

## 5. Inflammation, Stroma, and Increased Cell Survival

The phenotype of activated stromal fibroblasts is distinct from that of normal fibroblasts, including a more rapid proliferation rate and an increased survival rate [5,6,7]. In RA the recruitment and proliferation of inflammatory cells as well as the increased proliferation and survival of resident stromal cells promote synovial hyperplasia, neoangiogenesis and the attachment of the synovium to the adjacent cartilage and bone, ultimately resulting in joint destruction [18,19]. Factors such as TNF-α and IL-17 regulate the proliferation and survival of RASFs [15,134], promoting their accumulation in the hyperplastic synovium and at sites of invasion.

### 5.1. Proliferation vs. Apoptosis

NF-κB activation facilitates synovial hyperplasia by promoting proliferation and inhibiting apoptosis of RA synovial fibroblasts (RASFs) [135]. NF-κB is a positive regulator of cell growth in SFs primarily via the induction of c-Myc and cyclin D1, proteins required for cell cycle progression, but also via inhibition of the pro-apoptotic effects of c-Myc. Because c-Myc is highly expressed in RA synovium, NF-κB may thus contribute to hyperplasia, both inhibiting c-Myc induced apoptosis and promoting proliferation.

NF-κB also delivers an anti-apoptotic signal that counteracts other proapoptotic stimuli such as TNF-α, mediated by classical NF-κB activation. In the late stage of the arthritic process, the primary cell type in the pannus is the fibroblast cell that proliferates and secretes cytokines and enzymes in response to TNF-α. Although RASF cells have been demonstrated to express a variety of death inducing surface receptors of the TNF-α receptor family such as Fas/CD95 [136], TRAIL-R1 and -R2 [137,138] and also TNFR1 [139], multiple lines of evidence indicate that they are relatively resistant also to receptor induced apoptosis. Stimulation of cells with TNF-α has been shown to generate two signals, one that initiates programmed cell death and another that leads to the induction of inhibitors of apoptosis (IAPs) mediated by NF-κB activation and promotes the production of proinflammatory factors [37,140]. Thus, for instance, Fas can trigger both pro- and anti-apoptotic signals mediated by the expression of caspase 8 [141]. This process can be counterbalanced by the recruitment of FLICE-inhibitory protein (FLIP), which hetero-oligomerizes with caspase 8, leading to FLIP cleavage into the p43-FLIP form that induces the NF-κB and AP-1 proinflammatory pathways. Inhibition of either FLIP expression or caspase activity reduced Fas-induced proinflammatory signaling [141]. Similarly, inhibition of NF-κB nuclear translocation by gene transfer of dominant negative IκB [142] results in apoptosis in a variety of cell types originally resistant to TNF-α induced apoptosis [143,144]. Moreover, fibroblasts and macrophages from NF-κB subunit p65-deficient mice are more sensitive to TNF-induced apoptosis [145]. Adenovirus expressing high levels of a truncated form of IκBα that cannot be phosphorylated prevents nuclear translocation of NF-κB and leads to an unopposed apoptosis signal by RA synovial fibroblasts on culture with TNF-α [142,146]. Furthermore, recent observations at the cellular level have implicated aberrant expression of the Bcl-2 family members, myeloid cell leukemia 1 (Mcl-1) in the resistance of RA synovial fibroblasts to TNF-α- and Fas-mediated apoptosis [146,147]. Inhibition of Mcl-1 with epigallocatechin-3-gallate (EGCG), a potent antioxidant, antiinflammatory and antioncogenic compound, markedly inhibited the accumulation of p-Akt and the nuclear translocation and DNA binding activity of NF-κB p65 in TNF-α -stimulated RA synovial fibroblasts [148]. These data suggest that uncontrolled growth and induction of apoptosis in RA synovial fibroblasts is, at least in part, achieved by the inhibition of Akt and NF-κB signaling pathways.

In addition to the NF-κB transcription factor, STAT3 also has a relevant role in regulating the stromal proliferation and survival [149]. Accumulating data suggest that STAT3 has an anti-apoptotic effect that is linked to up-regulation of genes as Bcl2 and Bcl-xL, and down-regulation of Bax [150]. An imbalance between pro-apoptotic Bax and anti-apoptotic Bcl-2 exists in SFs from RA patients. In RASFs, inactivation of STAT3 by a dominant negative mutant induced apoptosis [149]. Constitutively activated STAT3-C (an STAT3 form dimerized by two cysteines instead of phosphotyrosine), protects fibroblasts from serum starvation-induced apoptosis [151]. *In vitro*, cultured RASFs expressed both IL-20R1 and IL-20R2, and induced IL-19 expression by LPS stimulation. Moreover, rIL-19 induced the production of IL-6, STAT3 activation and reduction of apoptosis. Therefore, IL-19 produced by synovial cells may promote inflammatory responses in RA synovial tissues by preventing cell apoptosis through STAT3 activation and the expression of IL-6. RASFs expressed both IL-19 and IL-20R complex consisting of IL-20R1 and IL-20R2, suggesting that IL-19 works in an autocrine as well as a paracrine manner [152]. Similarly, IL-17 can also modulate the Bcl2/Bax balance regulating RASF’s apoptosis. STAT3 mediated IL-17-induction of Bcl-2 and promoted the proliferation of synoviocytes, rescuing them from apoptotic death via STAT3 activation. In addition, pro-apoptotic Bax gene expression decreased in RASF cells, suggesting that the IL-17/STAT3 pathway is important for the survival and proliferation of synovial fibroblast [153].

Although in tumors, CAFs have been shown to proliferate faster than normal fibroblasts, the main feature that identify CAFs is their ability to promote cancer progression *in vivo*, usually when coinjected with tumor cells or when recruited to the tumor site [11,34,154]. Among the secreted factors that mediate the tumor promoting effect of CAFs is CXCL12 (SDF1), a chemokine that can induce angiogenesis and enhance the proliferative capacity of cancer cells [22]. Tumor growth factor β (TGF-β) signaling in fibroblasts has also been reported to modulate the oncogenic potential of adjacent epithelia [21]. These cytokines induced by inflammatory and stromal cells activate the expression of genes that stimulate cell proliferation and survival through transcription factors such as NF-κB, STAT3, and AP-1. Most of the genes that mediate the tumor-promoting functions of these transcription factors have not been fully defined, and most likely their protumorigenic effects are exerted through multiple effectors and similar to RASF cells, some targets may be controlled by more than one transcription factor and may be more important in one cell type than in another. Thus, for instance, the expression of the antiapoptotic proteins Bcl-2 and Bcl-XL is promoted by both NF-κB and STAT3, as is the expression of c-IAP1, c-IAP2, Mcl-1, c-FLIP, and survivin [29,155].

### 5.2. The Senescent Phenotype

Recent data have shown that CAF tumor-promoting activities are partially mediated through an altered expression profile that overlaps significantly with the senescence-associated secretory phenotype (SASP). Senescent cells undergo a stable cell cycle arrest controlled by RB and p53 and in addition, activate production of reactive oxygen species (ROS) [156] and the secretion of SASP [157,158,159], involving the production of factors that reinforce the senescence arrest, alter the microenvironment, and trigger immune surveillance of the senescent cells. The SASP is mainly transcriptionally regulated by NFκB but also by C/EBPβ [157,159,160]. Chronic, progressive low-grade inflammation induced by knockout mice that lack the expression of p105 and p50 NF-κB proteins induces premature ageing in mice. Both senescence-associated ROS [156] and NF-κB-driven pro-inflammatory cytokines, especially IL-6 and IL-8 [157,158], contribute to positive feedback loops that stabilize oncogene- or stress-induced senescence. Nfκb−/− fibroblasts from the chronic low-grade inflammatory mouse model exhibited aggravated cell senescence because of an enhanced autocrine and paracrine feedback through NF-κB, COX-2 and reactive oxygen species (ROS), which stabilizes DNA damage and cell senescence in the absence of any other genetic or environmental factor [161]. Chronic low-grade inflammation, similar to that observed in RA or cancer, promotes ROS-mediated DNA damage triggering telomere dysfunction and the increased accumulation of senescent cells, initiating a loop in which cell senescence aggravates chronic inflammation and limits tissue regeneration.

The SASP’s pro-tumorigenic nature has been demonstrated extensively both *in vitro* and *in vivo*. Senescent fibroblasts stimulate the invasiveness of human umbilical vascular endothelial cells (HUVECs) *in vitro* and increase vascularization of tumors in xenograft experiments through secretion of vascular endothelial growth factor (VEGF) [162]. Osteopontin (OPN) expression level is elevated in senescent fibroblasts and is necessary for the stimulation of preneoplastic cell growth induced by senescent fibroblasts *in vivo* [163]. Senescent human fibroblasts stimulate hyperproliferation and progression of preneoplastic epithelial cells and accelerated tumorigenesis by neoplastic epithelial cells. Treatment of co-cultures of senescence cells and preneoplastic epithelial cells with neutralizing antibodies against IL6 and IL8 results in decreased growth promotion [164]. Senescent human prostate fibroblasts stimulate the growth of epithelial cell harboring mutations that create preneoplastic cells in co-culture experiments while they have no effect on normal competent cells [165]. Finally, senescent fibroblasts also promote epithelial-to-mesenchymal transition (EMT) and invasion in breast preneoplastic cells [166], indicating the ability of senescent fibroblasts to promote not only the growth of preneoplastic cells, but also the progression from precancerous to cancerous lesions. It has been shown that treatment of human breast cancer cell lines with conditioned media from senescent fibroblasts resulted in decreased expression of cytokeratin and E-cadherin, the main feature of the EMT process [164]. This promotion of EMT by senescent cells was mediated by MMP3 [167]. These results demonstrate that senescent cells promote the establishment of primary tumors through the expression of the NF-κB-mediated SASP expression program [168].

## 6. Inflammation, Stroma, and Angiogenesis

The synovium shows an increased neoangiogenesis within the hyperplastic tissue, facilitating the influx of inflammatory cells [169]. During subsequent joint destruction, RASFs actively contribute to inflammation, angiogenesis and matrix degradation by producing inflammatory cytokines, proangiogenic factors and matrix degrading enzymes [18,19]. Local hypoxia in hyperplastic RA stimulates the expression by RASF cells of proangiogenic and chemotactic factors, matrix-degrading enzymes and osteoclastogenic factors among others, contributing to the joint destruction [18,19]. Hypoxia-inducible transcription factor (HIF)-1α and the hypoxia-induced expression of VEGF by RASFs are important factors that contribute to synovial neoangiogenesis and migration [16]. Other factors, such as angiogenin [170], angiopoietin-1 [171] and growth factors such as FGF-2 [172], are also produced by RASFs and contribute to vessel formation within the synovium.

Similar to what happens in arthritis, growth of large tumors requires an increased intratumoral blood supply mediated by tumor hypoxia, which promotes angiogenesis and increases the probability of metastasis. In addition to hypoxia, tumor angiogenesis depends on recruitment of tumor associated macrophages (TAMs) and stromal CAFs, which respond to hypoxic signals producing chemokines and proangiogenic factors. Recruitment of TAM precursors is largely dependent on the expression of angiogenic factors such as angiopoetin 2 and VEGF mediated, at least in part, by CAFs. Furthermore, CAFs have been shown to directly enhance tumor angiogenesis by either recruiting endothelial progenitor cells via their secretion of SDF-1/CXCL12 [22] or secreting proangiogenic factors [11,173]. Thus, skin CAFs enhanced angiogenesis in orthotopic squamous carcinoma tumors and in an *in vivo* Matrigel plug bioassay lacking cancer cells [11]. In these experiments, the upregulation of the proangiogenic gene CYR61 in skin HPV CAFs suggests that angiogenesis may be directly mediated, at least in part, by CAFs. In addition, CAFs-recruited macrophages are also proangiogenic in the HPV16-driven mouse models of skin and cervical cancers, through their secretion of MMP-9 and VEGF [174,175]. Thus, tumor angiogenesis may be mediated directly by CAFs or indirectly by macrophages recruited by the proinflammatory CAFs.

At a transcriptional level, important proangiogenic genes, such as IL-8, CXCL1, CXCL8, CXCL12, VEGF, and the HIF-1α transcription factor, are directly regulated by NF-κB, STAT3, AP-1, and HIF-1α itself, both in TAMs and CAFs, as well as other cell types [49,176]. Inactivation of NF-κB, STAT3, or HIF-1α, neutralization of CCL2 or CXCL12, or TAM depletion results in disrupted angiogenesis and decreased tumor growth, highlighting the critical role of inflammatory mediators in tumor angiogenesis [49,177].

A functional relationship has been demonstrated between HIF-1α and STAT3 signaling in the regulation of pro-inflammatory mechanisms. The link between HIF-1α and STAT3 occurs at different levels, and is further supported by studies showing that HIF-1α facilitates the binding of STAT3 to the haptoglobin promoter in HepG2 human hepatoma cells [178]. STAT3 also inhibits HIF-1α degradation through competition with Von Hippel-Lindau tumor suppressor (pVHL) for binding to HIF-1α, thus stabilising HIF-1α protein levels in tumor cells [179], and p-STAT3 is a potential regulator of HIF-1α-mediated VEGF expression in renal carcinoma cells [180]. This link between HIF-1α and STAT3 is also observed in RA. Hypoxia-induced cytokine production, cell migration and invasion in RASF cells were inhibited by siRNA STAT3 or the JAK2-inhibitor WP1066 [181]. The blockade of STAT3 signaling also inhibited hypoxia-induced HIF-1α and the expression of IL-6, IL-8, MMP3 and Notch-1 receptor mRNA in RA synovial tissue explants [181]. Owing to the central role of angiogenesis at sites of inflammation, therapeutic approaches have been discussed to target proangiogenic factors and the inhibition of HIF-1 α as candidate for potential therapeutic interventions [182].

## 7. Inflammation, Stroma and Invasion

From a clinical perspective, metastasis is the most critical aspect of tumorigenesis. Metastasis requires close collaboration between cancer cells, immune and inflammatory cells, and stromal elements. The stroma is a physical barrier that contains the tumor and prevents its spread. During the course of malignancy this barrier is breached by cancer cells that escape the tumor mass and are free to travel the bloodstream and colonize in distant sites. The first step is represented by epithelial-mesenchymal transition, in which cancer cells acquire fibroblastoid characteristics that increase their motility and allow them to invade epithelial linings membranes and reach efferent blood vessels or lymphatics [183]. Tumor cells then intravasate into blood vessels and lymphatics. Inflammation may promote this through production of mediators that increase vascular permeability. Some of these metastatic cells will be able to survive and travel throughout the circulation to finally interact, as single metastatic progenitors, with immune, inflammatory, and stromal cells and then start to proliferate [184].

### 7.1. The Epithelial-to-Mesenchymal Transition (EMT)

Already known factors controlling the epithelial-mesenchymal transition are Snail, a repressor of E-cadherin transcription in epithelial cells and TGFβ, which activates Smad transcription factors and MAPKs, regulating the expression of other modulators of the epithelial-mesenchymal transition, such as Slug [185]. An additional mechanism through which proinflammatory cytokines can affect the epithelial-mesenchymal transition is via STAT3-mediated induction of Twist transcription and NF-κB-mediated induction of both Twist and Kiss [29]. Twist basic helix-loop-helix transcription factor 1 (Twist1) is frequently overexpressed in stromal fibroblasts surrounding tumors as gastric cancer cells [186] and pharyngeal squamous cell carcinoma [187]. In addition, these Twist1-expressing stromal fibroblasts expressed CAF markers such as FSP1 and PDGFRα with association with poor prognosis [186]. IL6/STAT3 axis was discovered to be a key upstream control of Twist1, and IL6 was sufficient to induce Twist1 expression in normal fibroblasts and their transdifferentiation into CAFs via STAT3 phosphorylation. Microarray analysis of the effect of Twist1 on mRNA expression in fibroblasts identified CXCL12 as a key Twist1’s target in CAFs. Moreover, Twist1 was revealed to suppress cellular senescence of normal fibroblasts and CAFs.

Twist1 transcription factors may also be regulating the invasiveness ability of RASF cells. Using a computational network model, it was observed that Twist1, in addition to osteoblast-specific factor (POSTN), is a key regulatory candidate responsible for SF invasiveness. Interestingly, Twist1 and POSTN expressions were elevated in RASF and further upregulated by IL-1β. Furthermore, functional assays demonstrated the requirement of Twist1 and POSTN for migration and invasion of RASFs stimulated with IL-1β [188].

As mentioned above, the EMT is a very important event in tumor cell invasion and metastasis, and involves the loss of cell adhesion, cell-cell tight junctions, cell polarity, and remodeling of the cytoskeleton that facilitates cell migration and invasion [183]. Although fibroblasts do not undergo EMT themselves, EMT transformation-related markers such as E-cadherin, gp38, α-SMA and type IV collagen are expressed in RASF cells, in addition to their ability to migrate and invade cartilage at distant joints [189,190,191]. RASF exposed to hypoxic conditions increased cell migration and invasion, and the increase of HIF-1α expression and activation of Akt [192]. Upon knockdown or inhibition of HIF-1α in hypoxia by small interfering RNA or genistein treatment, the invasion ability of SFs was regained. HIF-1α was blocked with a phosphatidylinositol-3-kinase (PI3K) inhibitor indicating that HIF-1α activation was regulated by the PI3K/Akt pathway [192].

### 7.2. Extracellular Matrix Degradation and Cell Invasion

Cancer cell invasion requires extensive proteolysis of the extracellular matrix at the invasive front. Inflammatory cells are important sources of proteases that degrade the extracellular matrix. In a model of invasive colon cancer, CCR1+ myeloid cells, whose recruitment is triggered by the chemokine CCL9 produced by cancer cells, promote invasiveness through secretion of the matrix metalloproteinases MMP2 and MMP9 [193]. IL-1, TNF-α, and IL-6, promote MMP expression, invasiveness and metastasis via NF-κB and STAT3 [29]. On a broader scope, by altering the ECM composition, CAFs can influence tumor metastasis. Thus, the presence of intrametastatic αSMA-expressing cells appearing at the early stages of hepatic metastasis derived from B-16 melanoma cells in a mouse model was demonstrated [194]. The elevated presence of CAFs in metastatic human specimens was also evident by immunostaining in several types of cancer [195]. Both tumor cells and hematopoietic cells can activate CAFs in secondary sites to form niches for metastasis [196,197]. In these sites, CAFs “feed” the secondary tumor with secreted factors which support its growth [198]. CAFs are inherently equipped with motility and migratory capacities that can be exploited by tumor cells. In a 3D “organotypic” invasion assay, carcinoma cells used CAF characteristics in order to invade without the need to undergo EMT [199]. Another study suggests that under severe hypoxic conditions tumor cells elevate CXCR4, which allows them to migrate towards a gradient of CAF induced CXCL12 and escape to a normoxic environment at a distant site [200]. Overall, CAFs seem to be abundant at metastatic tumor sites and promote the transition of *in situ* tumors towards malignancy by affecting the rate-limiting steps of the process [4].

Migration and invasion of synovial fibroblasts (SFs) are critical in the pathogenesis of rheumatoid arthritis. RASF cells exhibit invasive characteristics reminiscent of cancer cells [14,191], such as reduced contact inhibition, reduced attachment-dependent growth [15] and, as already mentioned above, the ability to invade and “metastasize” *in vivo* [14,191], destroying cartilage and bone. Normal SF control the homeostasis of the ECM and synovial fluid, secreting a large variety of extracellular matrix components, enzymes able to destroy the ECM (such as MMPs), and inhibitors of matrix-degrading enzymes (such as tissue inhibitors of MMPs, TIMPS). Protease production synergizes with the high expression of adhesion molecules such as cadherin-11 to favour resorption of ECM and cartilage. The importance of SF in cartilage destruction was confirmed in mice with inflammatory arthritis, because animals deficient in cadherin-11 were protected from cartilage erosion [201].

SF are also putatively important promoters of bone erosion based on their ability to secrete the receptor activator of nuclear factor κB ligand (RANKL, also known as TNF ligand superfamily member 11), which promotes osteoclast differentiation [202]. SF invasiveness in patients with RA is partly stimulated by local proinflammatory factors such as IL-1 and TNF, reactive oxygen and nitrogen species whose formation is favored by local hypoxia, growth factors such as platelet derived growth factor (PDGF) and ECM proteins. However, invasiveness is also a feature retained by RASF, and can be detected in *ex vivo* invasion assays and in RASF-cartilage co-implantation assays in mice [14,203]. Thus, the invasive phenotype of SF in RA is dependent on both autonomous and local factors.

NF-κB transcription factor has important roles regulating the expression of these matrix metalloproteinases. In RASF cells, upon TNF-α and IL-1β induction NF-κB translocates into the nucleus and promotes increased transcription and secretion of MMP-3 and MMP-13 at least in part mediated by downregulation of the post-translational modifier SUMO2/3 [204,205]. Cadherin 11 engagement stimulates increased synthesis of several MMPs by RA synovial fibroblasts in a MAPK- and NF-κB-dependent manner [206]. Stimulation of RA synovial fibroblasts with Cad-11-Fc increased MMP-1 and MMP-3 at the protein and mRNA levels. It also increased the phosphorylation of the MAPKs JNK, ERK, and p38 kinase, the phosphorylation of NF-κB p65, and the nuclear translocation of activator protein 1 (AP-1) transcription factor. NF-κB and MAPK inhibitors partially blocked RASF MMP expression. NF-κB activity is also essential for upregulation of MMP-1 and MMP-3 in rabbit and human vascular smooth muscle cells [207].

Furthermore, the hypoxic environment, usual in the hyperplastic inflammatory processes, can enhance the effect of these cytokines. Thus, for instance, both hypoxia and interleukin-17A (IL-17A) promote the migration and invasion of fibroblast-like synoviocytes (SFs), which are critical for the pathogenesis of rheumatoid arthritis (RA) [208]. Stimulation of RASF cells with IL-17A under hypoxic conditions increased cell motility with no apparent epithelial-mesenchymal transition (EMT). Proinvasive effect of IL-17A on SFs under hypoxia may be mediated by the up-regulation of matrix metalloproteinase 2 (MMP2) and MMP9, induced by increased activation of NF-κB mediated by IL-17A and the activation of HIF-1α. Knockdown or inhibition of HIF-1α and NF-κB by small interfering RNA or specific small molecule inhibitors blocked IL-17A mediated and hypoxia-mediated MMP2 and MMP9 expression, cell migration, and invasion. The inhibition of NF-κB led to a marked decrease in the expression of HIF-1α, which indicated that IL-17A activated HIF-1α via the NF-κB pathway. These observations suggest a synergetic effect of IL-17A and hypoxia that might contribute to the migration and invasion of RASFs by upregulating the expression of MMP2 and MMP9 through activation of the NF-κB/HIF-1α pathway [208].

STAT3 is also a key transcription factor in RASF-mediated joint destruction in RA. STAT3 activation induced expression of receptor activator of nuclear factor kappa B ligand (RANKL), a cytokine essential for osteoclastogenesis [202], and STAT3 deficiency or pharmacological inhibition promoted significant reduction in expression of both IL-6 family cytokines and RANKL *in vitro*. STAT3 inhibition was also effective in treating the collagen-induced arthritis (CIA) RA model *in vivo* through significant reduction in expression of IL-6 family cytokines and RANKL, inhibiting both inflammation and joint destruction [209]. In this model, major inflammatory cytokines elevated in RA as IL-1β, TNF-α and IL-6, function in an amplification circuit for IL-6 family cytokines and RANKL via direct or indirect activation of STAT3. STAT3 activation further induced IL-6 family cytokines as well as RANKL, and lack of STAT3 abrogated both IL-6 family cytokine and RANKL expression. Pharmacological inhibition of STAT3 also inhibited expression of IL-6 family cytokines and RANKL in osteoblastic cells induced by IL-1β, TNF-α and IL-6 *in vitro* as well as in the joints of a CIA model *in vivo* [209].

## 8. Conclusions

In recent years, it has become clear that stromal cells are critical to the development of chronic inflammation in a variety of situations such as cancer or autoimmune diseases. Special efforts have focused on better understanding the fibroblast as a key player that sustains the production of pro-inflammatory mediators in the inflamed environment and, therefore, as a novel cell target for intervention in chronic inflammatory diseases and cancer.

In response to chronic inflammatory stimuli, fibroblasts undergo specific and context dependent phenotypic alterations which alter their pathogenic potential. Inflammatory fibroblasts have a different and pathological transcriptional pattern of expression compared with the normal cells from which they originate. The development of therapies targeting context-specific fibroblasts requires a deeper understanding of their transformation process.

Complex interactions between a relatively limited set of transcription factors regulate these patterns of gene expression upon chronic inflammation and play an essential role stabilizing the pathological phenotype of the stromal fibroblast. Therefore, a better understanding of this additional level of regulation from a drug discovery perspective may lead to the identification of new targets for the long-term modulation of chronic inflammatory diseases.
